# A mixed-methods, cross-sectional study of perceived stigma among Ugandans with epilepsy

**DOI:** 10.4314/ahs.v22i1.32

**Published:** 2022-03

**Authors:** Mark Kaddumukasa, Haddy Nalubwama, Carol Blixen, Nelson Sewankambo, Martha Sajatovic, Elly Katabira

**Affiliations:** 1 Makerere University College of Health Sciences, Medicine; 2 Mulago National Referral Hospital, Medicine; 3 Makerere University College of Health Sciences; 4 Neurological and Behavioural Outcomes Center, University Hospital Case Medical Center, Case Western Reserve University, Medicine

**Keywords:** Epilepsy, Stigma, Uganda

## Abstract

**Background:**

Epilepsy is associated with stigma and negatively impacts the lives of people living with epilepsy (PLWE) and their immediate families. More understanding of the stigma and discrimination experienced by PLWE in sub-Saharan Africa is needed.

**Methods:**

In a cross-sectional, mixed methods study, forty- eight PLWE who met the study inclusion criteria were enrolled. In depth interviews and focus group discussions were conducted and were audiotaped and transcribed verbatim. Analysis was conducted using a thematic, constant comparative approach with an emphasis on dominant themes. Perceived stigma was measured using the Kilifi epilepsy stigma score. Associations between socio-demographic factors and Kilifi epilepsy stigma score were assessed.

**Results:**

The median age of the study participants was 25 years, with median age (IQR) of epilepsy onset of 12 (6–18) years. The prevalence of high-perceived stigma was 31.9% (15/48). Seizure frequency was associated with high levels of perceived stigma (p-value of 0.038). Psychological abuse, rejections at home, places of employment and schools, poor relationships and intimacy and unmet engagements in social activities were cited as the perceived stigmatizing aspects among PLWE.

**Conclusion:**

In this Ugandan sample perceived stigma remains unacceptably high and interventions to address it are urgently needed in our settings.

## Introduction

Stigma in epilepsy remains a great challenge and is highly prevalent in sub-Saharan Africa 1. It negatively impacts the lives of people with epilepsy (PWE), their families and the communities where they reside 2. The occurrence of seizures within public places further worsens this stigma. Coexistence of psychiatric comorbidities, reduced physical and mental abilities, and social limitations may further exacerbate the stigma experienced 3. In sub-Saharan Africa, epilepsy is attributed to demonic possessions and highly believed to be a contagious disease 4–6. The fear of “catching the disease” delays medical assistance during seizure episodes and the thought that all epilepsy is hereditary, subsequently leads to resentments, ostracization and stigmatization. Misconceptions and poor knowledge regarding epilepsy play a big role in perpetuating epilepsy associated stigma. This subsequently results in an inability for PWEs to marry, inability to participate in community activities, attend school and negatively impacts their entire families 1,7–9. Though few cross-sectional studies within sub-Saharan Africa have been conducted, all have reported high levels of perceived stigma associated with epilepsy; Ethiopia (81%), Benin (69%), Nigeria (91.7%) and one qualitative study among adults in Tanzania 10–13. This study therefore set out to describe the perception of stigma by PWE within Uganda.

## Material and methods

The study was carried out at Mulago National referral and teaching hospital in Kampala, Uganda, between November 2017 and February 2018. Forty-eight (48) subjects attending the neurology and mental health clinics were consecutively enrolled into the study.

Two study sites at Mulago hospital were used to screen for study recruitment: the neurology outpatient clinic and mental health outpatient clinic. These clinics serve as a secondary and tertiary referral centers for patients with epilepsy in the districts surrounding the hospital.

### Study design

This was a mixed methods cross-sectional study design. Consecutive patients seen at the outpatient departments with a diagnosis of epilepsy (i.e., at least two unprovoked stereotyped afebrile seizures with eye witness corroboration with/without supportive inter ictal electroencephalographic findings, were approached for potential recruitment into the study.

Seizures were defined according to the International League against Epilepsy classification 14.

### Recruitment criteria

Patients aged 18 years and above with a clinical diagnosis of epilepsy, who were cognitively intact and gave written informed consent were recruited for the study. Patients who could not communicate, had mental retardation or dementia, with secondary epilepsy due to traumatic brain injury or stroke and had no attendant available were excluded. Of the 60 patients assessed during the study period, twelve were excluded (8 could not respond or communicate, 2 had mental retardation, and two objected to participate in the study due to conflicting schedules).

The study population consisted of 48 consecutive patients with epilepsy who met the inclusion criteria. For qualitative research, this sample size is within the recommended number of 20–50 individuals 15,16.

The Kilifi Stigma Scale of Epilepsy (KSSE), was used to quantify the perceived stigma experienced by PWE. It has been developed and validated for use in SSA 17. It utilizes a Likert score scale with 15 items each scored according to the participant's response that is score 0 for “Never”, score 1 for “Sometimes” and score 2 for “Always” (Table 2). It has a minimum total score of 0 and maximum of 30 which was calculated by summing up the score of all items. A total score of above the 66th percentile of the collected data indicated presence of high-perceived stigma, whereas that below indicated low-perceived stigma 18. Interviews were carried out in a secure safe room with only one participant (and their caretaker where necessary) at a time, to ensure privacy. Participants were given unique identification numbers to ensure confidentiality of their collected information.

**Table 2:** Responses by PWE as per each question in the KSSE Stigma Scale of Epilepsy

Number	Question / item	Not at all	Sometimes	Always
1.	Do you feel different from other people?	8	17	23
2.	Do you feel lonely?	11	20	17
3.	Do you feel embarrased?	9	13	26
4.	Do you feel dissapointed in yourself?	15	21	12
5.	Do you feel you cannot have a rewarding life?	11	24	13
6.	Do you feel you cannot contribute anything in society?	13	24	11
7.	Do you feel you cannot join others in public places?	12	16	20
8.	Do you feel other people are uncomfortable with you?	11	19	18
9.	Do you feel other people don't want to go to occasions with you?	13	19	16
10.	Do you feel other people treat you like an inferior person?	8	16	24
11.	Do you feel other people would prefer to avoid you?	11	18	19
12.	Do you feel other people avoid exchanging greetings with you?	13	20	15
13.	Do you feel you are mistreated by other people?	11	17	20
14.	Do you feel other people discriminate against you?	9	14	25
15.	Do you feel other people treat you like an outcast?	5	24	19
	**Total**	**160**	**282**	**278**

### Qualitative data collection and analysis

To optimize credibility and validity of results, both face-to-face in-depth interviews and focus group discussions (FGDs) were used to collect narrative data on perceived stigma 19,20. A semi-structured interview guide focused the discussion on these specific topic-related questions. For the qualitative study, the FGD topic-related questions have been used before in other similar studies especially in LMICs 13,21.

### Focus group methods

Six (6) separate Focus Group Discussions (FGDs) were conducted consisting of four groups with PLWE (n=19) and two with caregivers (n=8). An experienced qualitative researcher fluent in both English and Luganda (local language of the study area) moderated the FGDs. The interviews and FGDs explored participant views on epilepsy stigma as well as what they felt their community and care providers could do to reduce epilepsy associated stigma. All in depth interviews and FGDs were audiotaped, transcribed verbatim, and translated into English. The in-depth interviews' were conducted at the respondents' homes while the FGDs were conducted at the local village chairperson's residence. We conducted in-depth interviews in 12 PLWE previously discharged from Mulago national referral hospital in Kampala, and nine caregivers of PLWE. For the FGDs we selected individuals residing from nearby villages to enable easy movement and meeting.

The median age of PLWE was 24 years (IQR 19–30), with a range of 18 – 41 years, 10 (52.6%) were male. The median age of epilepsy onset was 12 years (IQR 6 – 18), range of 1 -37 years. Only one of the female PLWE was married, with 95% (18/19) unmarried. Two of the PLWE were students while 26% (5/19) were unemployed. The majority of PLWE (12/19) attained a secondary level of education.

The median age for caregivers was 50 years (IQR 45 – 50.5), with a range of 18–78 years. Seventy five percent of caregivers (6/8) were female. The majority of caregivers had some source of income or held a dedicated form of employment; only two (2/8) were unemployed.

### Qualitative data collection and analysis

Using both face-to-face in-depth interviews and FDGs, narrative data on perceived barriers to epilepsy care and coping with stigma were utilized to optimize credibility and validity of this qualitative study 19,20. We used open-ended questions so as not to limit the range or breadth of discussion among the participants. We positioned the study participants for adequate eye contact with others in the group and the discussion lasted approximately one hour.

Some of the examples of the open-ended questions used to explore the perceived stigma in epilepsy and epilepsy stigma reduction include; “*What sort of things get in the way of helping you in managing your epilepsy?” What is the impact of being diagnosed with epilepsy or seizure, social relations, coping with seizures, treatment, and resources sought. “What do you think the terms ‘seizure’ and ‘epilepsy’ mean to people in your community/culture?” The guide also included examples of follow-up probes such as “would you explain further”, “please describe what you mean”, and “would you give me an example*”. The interview was recorded, and then transcribed verbatim. Information collected from focus group sessions included interview and observation

A team of three investigators (MK, HN and MNK) read the focus group transcripts in their entirety to gain familiarity with the data. Segments of text from the transcript were labeled and assigned codes that described meaning of content^22^

The codes were subsequently collapsed into broad themes or categories. MK, HN and MNK independently coded each transcript to ensure consistency and transparency of the coding; discrepancies were resolved by discussion. Finalization of codes was based on the consensus of the qualitative team. We utilized the grounded theory approach to data analysis, involving open, axial and sequential coding, and the constant comparative method to generate constructs (themes) and elaborate the relationship among them 23. A separate coding dictionary was then constructed for the interviews and focus groups.

The focus groups and interview transcripts were coded and analyzed separately. Three qualitatively trained investigators (MK, HN and MNK) independently coded each transcript to ensure consistency and transparency of the coding; discrepancies were resolved by discussion. We used a grounded theory approach to data analysis, encompassing open, axial and sequential coding, and the constant comparative method to generate constructs (themes) and elaborate the relationship among them 23. A separate coding dictionary, that included mutually exclusive code definitions, was then constructed for the interviews and focus groups. The coding structure for each was reviewed after a preliminary analysis of a sub-sample of transcripts, and each dictionary was refined through comparison, categorization and discussion of each code's properties and dimensions^23^,^24^.

### For the quantitative study

A composite questionnaire comprising the patient's demographic data, disease-related variables such as date of initiation of AEDs and type of therapy (poly-therapy or mono-therapy) was used. The Kilifi Stigma Scale of Epilepsy (KSSE), was used to quantify the perceived stigma experienced by PWE. It has been developed and validated for use in SSA^17^. It is a Likert score scale with 15 items each scored according to the participant's response that is score 0 for “Never”, score 1 for “Sometimes” and score 2 for “Always” (Table 2). It has a minimum total score of 0 and maximum of 30 which was calculated by summing up the score of all items. A total score of above the 66^th^ percentile of the collected data indicated presence of high-perceived stigma, whereas that below indicated low-perceived stigma. Interviews were carried out in a secure safe room with only one participant (and their caretaker where necessary) at a time and the researcher to ensure privacy. Participants were given unique identification numbers to ensure confidentiality of their collected information.

### Statistical analysis

The socio-demographic and clinical variables were summarized using proportion and percentages for discrete variables and median for continuous variables. Spearman's rank correlation coefficient (r) was used to correlate epilepsy stigma score with socio-demographics. Associations with p values less than 5% were considered statistically significant. All analyses were performed using Stata version 14 (StataCorp.2015. Stata Statistical Software: Release 14, StataCorp LP, College Station, TX, USA).

## Results

### Demographic characteristics

Males comprised 56.3% (27/48) of the study participants. The median age in years (IQR) was 25 (19–34) years in PWE. Over half of the study participants were unemployed and 54.2% had attained up to a primary education. The majority (79%) of the participants were not married (38/48). The median perceived stigma score (IQR) was 18 (8–27) as shown in Table 1. Most PWE response to the KSSE was “sometimes” (282 responses), while 272 responded “always”. Majority of the respondents reported that they felt embarrassed, discriminated against and being treated as an inferior person as indicated and Table 2.

**Table 1:** Clinical characteristics of the study participants

**Clinical characteristic**	n	Median (IQR)
**Female sex**	21	43.8%
**Age onset of epilepsy in years**	48	12 (6 – 18)
**Seizure severity score**	48	28.5 (12 – 88)
**Epilepsy type**		
Generalized	40	83.3
Partial	8	16.7
**Seizure frequency/ episodes per year**		
No seizure	10	20.8
1–9	20	41.7
10–20	3	6.3
≥ 21	15	31.3
**Medication type**		
Monotherapy	5	11.1
Polytherapy	40	88.9
**Duration of epilepsy**		
<2 years	2	4.4
2 to <5 years	6	13.0
5 to <10 years	11	23.9
≥10 years	27	58.7
Kilifi Epilepsy Stigma Score	48	18 (8 – 27)

Overall, 31.9% (15/48) of the study participants reported high levels of perceived stigma, while 69.1% reported low levels of perceived stigma. Seizure frequency and female gender were associated with high levels of perceived stigma with p-values of 0.038 and 0.005 respectively. Other demographic and clinical factors were not associated with high levels of perceived stigma, see Tables 3 and 4.

**Table 3:** Association between demographic characteristics and level of perceived stigmaƗ

	High perceived stigma n (%)	Low perceived stigma n (%)	p value
**Overall**	15 (31.9)	32 (69.1)	
**Age onset of epilepsy in years**			0.172
< 10 years	9 (60.0)	9 (28.1)	
10 – 19 years	5 (33.3)	16 (50.0)	
20 – 29 years	1 (6.7)	4 (12.5)	
≥ 30 years	0 (0.0)	3 (9.4)	
**Current age in years**			
10 – 19 years	3 (20.0)	9 (28.1)	0.858
20 – 29 years	6 (40.0)	10 (31.3)	
≥ 30 years	6 (40.0)	13 (40.6)	
**Sex**			0.038*
Male	5 (33.3)	21 (65.6)	
Female	10 (66.7)	11 (34.4)	
**District, n (%)**			0.233
Mukono	11 (73.3)	14 (43.8)	
Kampala	2 (13.3)	11 (34.4)	
Wakiso	2 (13.3)	4 (12.5)	
Others	0 (0.0)	3 (9.4)	
**Marital status, n (%)**			0.368
Single	14 (93.3)	23 (71.9)	
Married	1 (6.7)	6 (18.8)	
Divorced	0 (0.0)	3 (9.4)	
**Employment status, n (%)**			
Employed	4 (26.7)	16 (50.0)	0.206
Unemployed	11 (73.3)	16 (50.0)	
**Education status, n (%)**			0.512
None	1 (6.7)	0 (0.0)	
Primary	8 (53.3)	16 (50.0)	
Secondary	6 (40.0)	14 (43.8)	
University	0 (0.0)	2 (6.3)	

Ɨ Total score of above the 66th percentile considered to be high-perceived stigma

**Table 4:** Association between clinical characteristics and level of perceived stigmaƗ

	High perceived stigma N=15	Low perceived stigma N=32	p value
**Seizure severity score**, median (IQR)	74 (10–163)	26 (12–69.5)	0.278
**Epilepsy type**, n (%)			0.697
Generalized	12 (80.0)	27 (84.4)	
Partial	3 (20.0)	5 (15.6)	
**Seizure frequency/ episodes per** **year, n (%)**			0.005
No seizure	2 (13.3)	8 (25.0)	
1–9	2 (13.3)	17 (53.1)	
10–20	2 (13.3)	1 (3.1)	
≥ 21	9 (60.0)	6 (18.8)	
**Medication type**, n (%)			>0.999
Monotherapy	2 (13.3)	3 (10.3)	
Polytherapy	13 (86.7)	26 (89.7)	
**Duration of epilepsy**, n (%)			0.142
<2 years	1 (6.7)	1 (3.3)	
2 to <5 years	1 (6.7)	4 (13.3)	
5 to <10 years	1 (6.7)	10 (33.3)	
≥10 years	12 (80.0)	15 (50.0)	

Ɨ Total score of above the 66th percentile considered to be high-perceived stigma

### Qualitative study

Transcript-based analysis generated 5 major domains reflecting the issues of perceived or enacted stigma that our PWE respondents faced in relation to having epilepsy: (1) psychological abuse, (2) rejection, (3) infringements on personal rights 4) poor relationships or intimacy, and 5) unmet engagements in social activities. The illustrative quotes emerging from the discussion regarding epilepsy-associated stigma are presented in Table 5.

**Table 5:** Illustrative quotes from the study respondents

Themes and Categories	Illustrative Quotations from Respondents
**Verbal abuse or** **demeaning insults**	*“I would get attacks from class and my fellow students would* *laugh at me. This was stigmatizing” Respondent PR #12*
	* “My own father sees me negatively and calls me a psychic. He* *says, “I wouldn't want a psychic like you to work with other* *people. How can you imagine yourself working with other sane* *people?” Respondent PR #5*
**Isolation and** **avoidance**	*“They say, you have epilepsy so don't touch my stuff; and they* *therefore find you disgusting and they isolate you”. Respondent* *PR#4* *“I have siblings but because of this illness; they act distant and* *don't love me” Respondent PR#15.*
**Lack of power to** **make personal** **decisions.**	*“My father asked me to quit school. He finally made the* *decision. However, I have some hope of resuming school”.* *Respondent PR# 3:*
**Limitations on** **engagements in** **social activities.**	*“We know you have a problem but we don't expect you to do* *this and that or move here and there,” so I have to just sit there* *while my colleagues are working” Respondent PR #6*
**Poor relationships** **and intimacy.**	*“I have a girlfriend but I wouldn't want her to find out about* *my disease. I may fail to get another one who will understand* *my condition” Respondent PR #1*

### Psychological abuse

Many of the study participants described psychological abuse through verbal abuse, mockery, name -calling, laughed at, stared at, gossiped about and demeaning insults by their immediate family and community members due to their disease.

“*Even in the community they call me a mentally ill person. Despite knowing me by my name, they say, “we know about her brain” Respondent PR#7*

*“This illness, wherever you pass, they never call you by your name anymore. There's also a word that they use although I don't want to use it, “epileptic”. Everywhere you pass they say, “That one is an epileptic! That is the most hurtful thing*”. Respondent PR#2.

### Rejection

The study participants reported rejection through social isolation and avoidance by individuals at their homes, schools and places of work because of their illness.

#### a Rejection at schools

One of the study participants shared her experience of being rejected and isolated at school due to misconceptions of infecting others. “*Even when we are at school, I can get just one attack and then my colleagues isolate me. I have a friend who used to help me, she is no longer sharing a seat with me anymore, and they say that when they sit with me I would infect them with epilepsy. Now I sit in my corner alone*” Respondent PR#10

#### b Rejection at employment stations

Some of the respondents reported mentioned not being hired, associated with while at their work stations or being denied jobs they previously held before diagnosis of epilepsy. “*They (colleagues and bosses) told me that they didn't want a mentally unstable person, because I was having frequent attacks, they said that am a burden to them. They switched me to start working outdoors alone*”. Respondent PR#7:

#### c Rejection at home

The study respondents reported being restricted, isolated and rejected by their immediate family members was encountered by some respondents. “*I can never sit close to some people; he can even push you away. If you are chatting in a group he can even say, “That one should leave because he doesn't fit into our group!*” Respondent PR#12:

### Infringements on personal rights.

Some of the study participants felt that there was reported infringements on their rights to make personal decisions which renders them incapacitated. The power of decision making was relinquished to the caregivers/parents. “*What has been my biggest challenge is that in most cases the people you live with make decisions for you. I was interested in education, but I would get attacks from class and other students would laugh at me. Despite that I was performing well at school but they told me, “you need to stop studying; go back home.” They (teachers) told my parents that they should discontinue me from studying so I stopped at that stage*”. Respondent PR#2:

Unmet engagements in social activities and networks

Feelings of shame, insecurity and low esteem associated with having a seizure in a social gathering or event made some of the study participants reported fear to engage or participate in social events or activities within their communities. They thus coped by adopting solitary life styles due to their disease or fear of having a seizure. They reported feeling insecure, ashamed about themselves and having a low self-esteem. “*I don't want to spend a lot of time where many people are because what if I got an attack and they see me?” So I always move alone or spending my time sleeping in the house*”. Respondent PR#3.

### Poor relationships and intimacy

Poor relationships and intimacy with friends and spouses were experienced by some participants once their loved ones got to know about their disease condition.

“*It is that I had a boyfriend but because he got to know my illness we separated*”. Respondent PR#4:

### Reported consequences of the perceived or enacted stigma to PWE

#### 1 Unmet employment expectations and restrictions

The study participants noted that finding and maintaining jobs or employment was crucial for PWE as they require an income to cater for their out of pocket purchases of anti-epileptic drugs as well as taking care of their personal and family needs. However, PWE were concerned about society's perceptions about the condition, which adversely affected many PWE's chances of securing and maintaining jobs.

“*It has affected the work that I do when someone like your employer sees you having an attack from the job, he says, “I won't let him die from here.” So you get dismissed from that job*”. Respondent PR# 16:

“*The biggest is work; there are certain kinds of jobs that you can never do anymore. For example riding a boda-boda (motorcycle), roofing a house if you were a builder, you would have to quit such kinds of work. Well you might feel like you can handle but your employer wouldn't let you., “I can't let you die from my work*.” Respondent PR#1

#### 2 Low self-esteem among PWE

Study respondents' expressed periods of feelings of being unwanted and feelings of inferiority among people around them. “The illness makes you feel unwanted or feel like you are more unattractive than people around you. So you kind of feel jealous which gets you stressed and consequently get an attack”. Respondent PR# 2

“*They said that I have had epilepsy for so long and they say that if you have it for so long you run mad.” That hurt me so much and makes me feel small to my friends*”. Respondent PR#8

#### 3 Lost social roles and opportunities

The respondents related that they had lost social responsibilities, roles and opportunities by not being employed, not attending school, not having intimate relationships or getting married, not allowed to participate in household chores, or to participate in religious activities and taking care of their families.

“*She must not cook. She should never cook for us! What if she falls into the food? She should not go close to fire*.” Respondent PR#3

“*I feel bad when I wake up early in the morning and I see people go to work while I'm seated at home, I keep saying every day I'm like, this thing doesn't heal I'm like an HIV sufferer*” Respondent PR#18

#### 4 Unfulfilled dreams and expectations

Study respondents expressed shattered dreams and criticisms from family members regarding their disabilities. “*This illness hindered me, otherwise I was so bright. In fact, I was going for a nursing course but it (disease) hindered me. I was getting the attacks every day! Consequently I realized that it was hard to continue with school, I couldn't study it with that illness*”. Respondent PR#2

#### 5 Having suicidal tendencies

Some of the respondents revealed that at some point they felt so low that they even thought of killing themselves during the course of their illness. I asked myself “*Why am I even suffering in this world? Why don't I kill myself?” I now don't have such suicidal thoughts anymore*”. Respondent PR#15

“*Yes I was feeling like I was fed up of the world so I thought, “should I just take my life?” But seeing other people living with epilepsy and also knowing that epilepsy can be cured gives me hope*”. Respondent PR#16

#### 6 Financial strain

The respondents reported increased financial strain due to unmet employment opportunities as well as providing for their families was noted as a major challenge encountered by some study participants. “*My biggest challenge is that the doctors tell us not to overwork and yet I have to work. Sometimes I run out of pills and yet I don't have money to come for more drugs so I skip my medication for days*”. Respondent PR#4

Management of the epilepsy – related stigma by PWE. The study participants described various ways in which they managed the perceived and enacted stigma. Some of the study participants reported concealing their illness, ignoring the offending comments made and resorted to participating in religious activities.

“*What has enabled me to work is that people in my community don't know about my illness, I pray I do not get a seizure while at work*”. Respondent PR#16

Others reported having a positive outlook and acceptance of the condition and medication.

“*Personally, I accepted the illness and also turned swallowing medication part of my daily activities, this has helped me to remain seizure free for some time*”. Respondent PR# 14

“*With the kind of illness that we have, you must know what you are. I am now free because my friends and people at home know about it and we always move together.*” Respondent PR#1

“*For that stigma to end you need to accept who you are. I was at school finishing with my senior six; I suddenly got an attack and the entire school got to know about it since we were at the assembly. So that was the time when I said, “It is high time I accepted what I am; I am not afraid anymore*”. Respondent PR#11

Some PWE reported that they participate in religious activities and trust in God as well as associating with people less likely to stigmatize them.

“*Personally, I never get stressed because the bible gives you a gift of self-control*”. Respondent PR#2

Other study respondents reported downplaying derogatory comments regarding their illness.

“*You ignore the comments and relax or take a nap. Respondent PR #9*

*“There's nothing I do; I wait until I get something to calm me down. Sometimes I watch a movie and think over it*”. Respondent PR #8

## Discussion

This study set out to explore the perceived stigma in people with epilepsy. The study findings indicate that PWE in Uganda report diverse views on perceived stigma.

In Uganda, PWE experience stigma and reported that it negatively impacts on their social networks, roles and opportunities in society, financial burden, and mental health. This was reported in various domains which included psychological, rejection, rights and relationships with friends, family, or the community as a whole and reported to be stigmatized due to their disease. The social stress theory emphasizes the fact that the anticipation of negative treatment and the accompanying chronic stress results in a permanent state of vigilance^25^. In this situation, PWE experience perceived stigma tend to intensify the stressful circumstances and compromise on their ability to cope with these circumstances^25^,^26^. Therefore, PWE approach interactions in society with concern. Work by Goffman shows that stigma casts a long shadow and has the potential to impact those who are stigmatized^27^. This is further worsened by social processes which perpetuate separation and isolation of the stigmatized individual with loss of social status and discrimination. Stigmatization in epilepsy that is empowered by social, cultural, economic and political aspects subsequently leads to unequal health and poor socioeconomic outcomes 28.

In Uganda and the rest of SSA, the belief that epilepsy is heritable is prevalent^5^,^13^ and this belief is thought to be a significant factor in the poor relationships and intimacy. In this study, we did not explore the differences between genders. The feelings of shame, insecurity and low esteem all impact on the social networks and interactions that would help establish relationships and intimacy. This is one of the main reasons for failure to disclose their disease condition. The study reports that PWE actually fear when their disease condition is disclosed to others. Sometimes, they report that they have denied clearly that they have epilepsy. This non-disclosure subsequently tends to delay seeking appropriate health care with majority preferring traditional care leading to the treatment gap^29^. However, by refusing to discuss their disease, PWE may be limiting their opportunity for receiving supportive social relationships when needed. This lack of a social network can influence health outcomes, leading to a lower overall quality of life.

Participants in our study who had higher seizure frequencies were associated with higher perceived stigma. These results were consistent with earlier studies that have reported that PLWE who experience seizures report higher levels of stigma 12,30,31. The associated seizure worry, physical and psychological trauma may immensely contribute to perceived stigma if the seizures are not controlled. The perception of poor seizure control and negative seizure outcomes were reported to predict perceived stigma 32. Female gender in our population had higher levels of perceived stigma in our sample. It was difficult to understand whether the effect is not due to the seizure frequency as these were comparable between male and females in our study. This may be explained by the patriarchal system in Uganda that provides more tolerability to men's problems as compared to women. This cultural - societal trend engulfs all aspect of life such as health, employments, responsibilities and decision making. Secondly, generally females have greater level of psychological distress in comparison to the males. So they are prone to experience greater level of discrimination and stigma then Men 33,34.

In this study, social factors (social isolation and rejection, issues with relationships) appear to be the main aspects influencing stigma in this population. Nevertheless, poor social function in PWE was prevalently reported in this sample. The majority of the study participants were unemployed which they attributed to their illness. Unemployment remains a major concern and problem for people with epilepsy due to the various misconceptions and prejudices about people with epilepsy. PWE reported discrimination, receiving fewer workplace rewards and they are more likely to receive job terminations than other employees. The current social and legal trends worldwide aim to combat discrimination against people with medical disabilities such as epilepsy and bring down barriers to employment. Advocating for better working terms and conditions especially for those whose seizures are under control will help address these challenges.

Finally, addressing stigma towards people with epilepsy requires a multidisciplinary and multipronged effort to address stigma at different levels in sub-Saharan Africa. Utilizing highly successful public health models that have been successfully utilized to tackle HIV stigma in Uganda, we would adopt them to help reduce the burden of epilepsy associated stigma.

## Limitations

Our study findings on perceived stigma to epilepsy care in PLWE in Uganda have implications for informing policy and care. Notwithstanding, there were some limitations that need to be taken into account. Patients with epilepsy who receive or seek care in other settings in Uganda, may have different experiences with, and different types of encounters with providers or healthcare systems from our study. The small convenience sample utilized in this study and the conduct of the study in a single urban area in Uganda may limit transferability of the study findings. In addition, seizure frequency was self-reported and the seizure frequency classifications were determined arbitrarily and treatment adherence was not explored. However, these limitations are offset, to some extent, by the utilization of rigorous qualitative methods described in the study and our use of the Consolidated Criteria for Reporting Qualitative research (COREQ)^35^, to improve the rigor, comprehensiveness and credibility of the interviews and focus groups.

## Conclusion

This study found that PWE experience stigma which negatively impacts on their social networks, roles and opportunities in society, financial burden, and mental health. Developing culturally feasible strategies to reduce stigma in our setting would be an important measure in epilepsy care in Uganda.

## Declarations

### Availability of data and material

All data generated or analyzed during this study are included in this published article.
